# Mechanism of LDH and IL-8 involved in pancreatic cancer pain and the correlation of pain degree

**DOI:** 10.5937/jomb0-48160

**Published:** 2024-09-06

**Authors:** Zhiming Zhou, Zongfeng Guo, Xiaomin Lu, Xiaoqing Xu

**Affiliations:** 1 Nantong University, Affiliated Haian Hospital, Department of Interventional Oncology, Nantong, China; 2 Nantong University, Affiliated Haian Hospital, Department of Anesthesiology, Nantong, China; 3 Nantong University, Affiliated Haian Hospital, Department of Oncology Pain, Nantong, China

**Keywords:** lactate dehydrogenase, interleukin 8, pain, pancreatic cancer, mechanism, laktat dehidrogenaza, interleukin 8, bol, kancer pankreasa, mehanizam

## Abstract

**Background:**

This research aimed to observe the mechanism of lactate dehydrogenase (LDH) and interleukin 8 (IL8) in pancreatic cancer pain and their correlation with pain degree.

**Methods:**

126 patients with pancreatic cancer who visited our hospital from January 2021 to February 2023 were selected. The patients were divided into groups of 58 patients with low pain (1~3 points) and 68 patients with high pain (4~10 points) by visual analog scale (VAS). And 50 health examinees in the same period were selected as the healthy control group. The serum LDH and IL-8 concentrations are analyzed by enzyme-linked immunosorbent assay, and the subjective pain grading method score is analyzed. The differences in LDH and IL-8 concentrations among the three groups of patients were compared. Pearson correlation analysis was used to investigate the correlation between LDH, IL-8 concentrations, and patient pain. Binary logistic regression was used to determine independent risk factors for high pain, and ROC curves were used to analyze the diagnostic efficacy of each indicator.

**Results:**

The serum LDH and IL-8 concentrations in the high-pain group were exceed the low-pain group's (P<0.05). The serum LDH and IL-8 concentrations in the low-pain group exceeded the healthy control group's (P<0.05). Pearson correlation analysis revealed a positive correlation between serum LDH concentration and pain grading (r=0.736, P=0.000). The serum IL-8 positively correlates with pain grading (r=0.680, P=0.000). Serum LDH and IL-8 concentrations positively correlate (r=0.589, P=0.000). LDH and IL-8 concentrations are independent risk factors for high pain levels (OR=1.033, 1.142, P<0.05). The logistic regression prediction model formula was used: Y=constant+B1X1+B2X2+...+BnXn to set the joint diagnostic prediction model as -12.063+ 0.033×LDH+0.133×IL-8. The areas under the ROC curves of LDH, IL-8, and predictive model (LDH+IL-8) in patients with high pain were 0.925, 0.945, and 0.974, respectively. The relevant standards for LDH are >190 U/L, IL-8 is >36 pg/mL, and the relevant standards for prediction models are >5.75.

**Conclusions:**

LDH and IL-8 participate in the pain aggravation process of pancreatic cancer and are closely related to the pain grading. The combination of LDH and IL-8 can be used as a biological indicator to evaluate the pain severity of pancreatic cancer and provide a reference for clinical diagnosis and treatment.

## Abstract (sr)

**Metode:**

Odabrano je 126 pacijenata sa kancerom pankreasa koji su posetili našu bolnicu od januara 2021. do februara 2023. Pacijenti su podeljeni u grupu od 58 pacijenata sa niskim bolom (1~3 poena) i grupu od 68 pacijenata sa jakim bolovima (4~10 poena) prema vizuelnoj analognoj skali (VAS). Kao kontrolna grupa odabrano je 50 zdravih ispitanika u istom periodu. Koncentracije LDH i IL-8 u serumu su analizirane enzimskim imunološkim testom, a ocenjena je i subjektivna metoda procene bola. Upoređene su razlike u koncentracijama LDH i IL-8 između tri grupe pacijenata. Pearsonova korelaciona analiza je korišćena za istraživanje veze između koncentracija LDH, IL-8 i stepena bola. Za utvrđivanje nezavisnih faktora rizika za visok bol korišćena je Binarna logistička regresija, a ROC krive su korišćene za analizu dijagnostičke efikasnosti svakog pokazatelja.

**Rezultati:**

Koncentracije LDH i IL-8 u serumu u grupi sa visokim bolom su premašile koncentracije u grupi sa niskim bolom (P<0,05). Koncentracije LDH i IL-8 u grupi sa niskim bolom su bile više nego kod zdrave kontrolne grupe (P<0,05). Pearsonova korelaciona analiza je otkrila pozitivnu korelaciju između koncentracije LDH u serumu i ocene bola (r=0,736, P=0,000). IL-8 u serumu je u pozitivnoj korelaciji sa ocenom bola (r=0,680, P=0,000). Koncentracije LDH i IL-8 u serumu su u pozitivnoj korelaciji (r=0,589, P=0,000). LDH i IL-8 su nezavisni faktori rizika za visok stepen bola (OR=1,033, 1,142, P<0,05). Formula logističke regresije je korišćena za predviđanje modela: Y=konstanta+B1X1+B2X2+...+BnXn postavljajući zajednički dijagnostički model kao -12,063+ 0,033×LDH+0,133×IL-8. Površine ispod ROC krivih za LDH, IL-8 i prediktivni model (LDH+IL-8) kod pacijenata s visokim bolom su bile 0,925, 0,945 i 0,974, redom. Relevantni standardi za LDH su >190 U/L, za IL-8 >36 pg/mL, a za prediktivne modele su >5,75.

**Zaključak:**

LDH i IL-8 učestvuju u procesu pogoršanja bola kod kancera pankreasa i tesno su povezani sa ocenom bola. Kombinacija LDH i IL-8 se može koristiti kao biološki pokazatelj za procenu ozbiljnosti bola kod kancera pankreasa i mogu da daju referencu za kliničku dijagnozu i lečenje.

## Introduction

Pancreatic cancer (PC) is a common malignant tumor of the digestive system, and its incidence rateis on the rise [1]. According to statistics, there were approximately 495000 new cases of PC and approximately 420,000 deaths worldwide in 2020. In China, the 5-year relative survival rate of PCs is only 7.2% [2] [3]. The main clinical manifestation of PC is abdominal pain. Severe cancer pain can have adverse effects on PC patients’ life quality and is the main reason why PC patients seek medical help. Accurately determining the type and mechanism of pain is crucial for developing effective analgesic treatment plans. The research on the mechanism of PC pain mainly starts from multiple aspects, such as the tumor itself, inflammatory response, changes in the nervous system, and signaling pathways [4]. At present, the MAPK pathway [5], PI3K/AKT pathway [6], and COX-2 pathway [7] play important roles in PC hyperalgesia and neuropathic pain. Lactate dehydrogenase (LDH) and interleukin-8 (IL-8) are important biomarkers involved in the occurrence and development of tumors. LDH promotes the glycolysis of PC cells by providing them with energy substances, thereby promoting tumor growth. IL-8 participates in tumor progression by promoting PC cell proliferation, angiogenesis, and infiltration. LDH and IL-8 also participate in the mechanism of PC pain and are connected to pain severity. This study’s main hypothesis is that serum LDH and IL-8 concentrations significantly correlate with the degree of PC pain, which can serve as predictive factors for distinguishing different degrees of pain. To verify this hypothesis, correlation studies and ROC curve analysis were used in the experiment to determine the mechanism of action of LDH and IL-8 in PC pain. At the same time, the correlation between two biomarkers and pain severity was analyzed in the experiment. The research results can provide new ideas and directions for PC patients’ diagnosis, pain assessment, and analgesic treatment.

## Materials and methods

### Patients

126 PC patients who visited our hospital from January 2021 to February 2023 were selected, including 58 patients in the low-pain group (LPG) and 68 in the high-pain group (HPG). 50 healthy examinees were treated as the healthy control group.

Inclusion criteria: 1) Diagnosed as a PC patient through preoperative imaging examination. 2) 18–75 years old. 3) Karnofsky score 70, estimated survival time 3 months. 4) The patient feels pain and is able to cooperate in assessing the degree of pain.

Exclusion criteria: 1) Combined with other malignant tumors or life-threatening diseases such as severe organ failure. 2) Mental illness or inability to cooperate with research. 3) Within the past month, drugs such as immunosuppressants and glucocorticoids have been used. 4) Blood system diseases or coagulation disorders. 5) Regular follow-up or missing visits during the research process cannot be guaranteed. 6) Postoperative pathology confirmed a non-PC patient.

The health control group consists of health examination personnel aged 18–75 without any physical pain sensation. LPG has 34 males and 24 females, with an average age of (63.5±12.3), ranging from 29 to 88 years. There were 32 cases with a history of drinking alcohol. A total of 42 males and 26 females were included in the HPG, aged 44–87, averaging (65.2±10.7) years old. 38 cases have an alcohol consumption history. In the healthy controlling group, 28 males and 22 females, averaging 64.3±8.5, aged 48–82. There were 28 cases with a history of alcohol consumption. The three groups’ gender, age, and alcohol consumption history are the same (*P*>0.05).

### Research methods

### General information survey

A general information questionnaire was selfmade. Two scale investigators conducted information collection in the patient’s ward. The collection content includes gender, age, smoking history, drinking history, tumor staging, chemotherapy history, and targeted treatment history.

### Pain grading method

Pain assessment was conducted based on the visual analog scale (VAS) score of patient pain. A linear table with a length of nearly a certain length (10 cm) was labeled as »painless« and »most severe pain,« representing their respective endpoints. The patient is required to indicate the current level of pain felt on this straight line. VAS score was obtained by measuring and recording the distance indicated by the patient in the experiment. The range of values is 0–10, where 0 represents painless, and 10 represents the most severe pain. 1–3 points are considered mild and included in the LPG, 4–6 points are classified as moderate pain, 7–10 points are classified as severe pain, and 4–10 points are included in the HPG.

### LDH and IL-8 concentration determination

Five mL of venous whole blood samples from patients were collected in the experiment, and theserum was separated by conventional centrifugation and stored at 4°C. LDH was quantitatively detected by enzyme-linked immunosorbent assay (ELISA). First, an LDH ELISA kit was prepared, including LDH antibody, LDH standard, biotinylation LDH secondary antibody, antibiotinase, a substrate solution (including TMB), and a termination solution pre-coated on the microplate. The serum sample to be tested and the LDH standard were diluted to an appropriate concentrationand added to a microporous plate pre-coated with LDH antibodies. The reaction was sustained for 30 minutes at 37°C. The board was washed 5 times to remove unbound substances. This experiment added biotinylation LDH secondary antibody, reacting at 37°C for 30 min. The board was washed 5 times to remove unbound substances. Antibiotinase was mixed in the experiment, and the reaction was sustained for 30 minutes at 37°C. The board was washed 5 times to remove unbound substances. Substrate solution (TMB) was added to the experiment, and the room temperature was dark and colored for 10 to 30 minutes. A termination solution was mixed in the experiment, and its absorbance at 450 nm was tested. The LDH concentration in the sample was calculated on the foundation of the standard curve. ELISA was used to detect serum IL-8 concentration quantitatively. First, an IL-8 ELISA kit was prepared for the experiment, including a microplate precoated with IL-8 antibody, IL-8 standard, biotinylation IL-8 secondary antibody, antibiotinase, a substrate solution (containing TMB), and a termination solution. Then, the serum sample to be tested and the IL-8 standard were added to the microplate at an appropriatedilution ratio in the experiment, and the reaction was sustained for 2 hours at 37°C. The board was washed 5 times to remove unbound substances. Biotinylation IL-8 secondary antibody was added, and the reaction was sustained for 1 hour at 37°C. The board was washed 5 times to removeunbound substances. Antibiotinase was added in the experiment, and the reaction was sustained for 30 minutes at 37°C. The board was washed 5 times to remove unbound substances. Substrate solution (TMB) was added to the experiment, and the room temperature was dark and colored for 30 minutes. A termination solution was mixed, and its absorbance at 450 nm was tested. IL-8 in the sample was calculated based on the standard curve.

### Observation indexes

(1) Comparison of general information. The differences in gender, age, smoking history, drinking history, Karnofsky score, tumor staging, chemotherapy history, and targeted treatment history among three groups of patients were compared. (2) Pain severity. The pain grading of PC patients was recorded, and they were classified as mild to moderate to severe pain patients. (3) Serological indicators. The LDH and IL-8 concentration measurement results of each group of patients were collected.

### Statistical methods

SPSS 24.0 is the analyzing tool. The measurement data belonging to the normal distribution were represented by x̄±s. Otherwise, they are represented by the median (interquartile range). ANOVA analysis of variance or Mann-Whitney testing was used. These counting data were represented by example (%). χ^2^ test was used. Pearson correlation analysis can identify whether LDH, IL-8 concentration, and patients’ pain degree have a correlation. Binary logistic regression can identify independent risk factors resulting in high pain, and a joint diagnostic prediction model was constructed. The receiver operating curve (ROC) can analyze LDH, IL-8, and predictive models for high pain severity. Medcalc software determined the relevant standards for each data for high pain severity, and the correction level a was set at 0.05.

## Results

### Clinical data comparison in two groups

In Table 1, there was no statistical significance in tumor staging, chemotherapy history, and HPG and LPG’s targeted therapy history are the same (*P*>0.05).

**Table 1:** Clinical data comparison.

Group (n)	Tumor staging<br>(case)		History of chemotherapy<br>(case)	History of targeted therapy<br>(case)
	I/ stage	III/ stage		
Low pain (58)	12 (20.69)	46 (79.31)	38 (65.52)	26 (44.83)
High pain (68)	9 (13.24)	59 (86.76)	54 (79.41)	37 (54.41)
*t*/χ^2^	1.252		2.408	1.150
*P*	0.263		0.121	0.284

### Comparison of serum LDH and IL-8 concentrations among three groups

**Figure 1:**
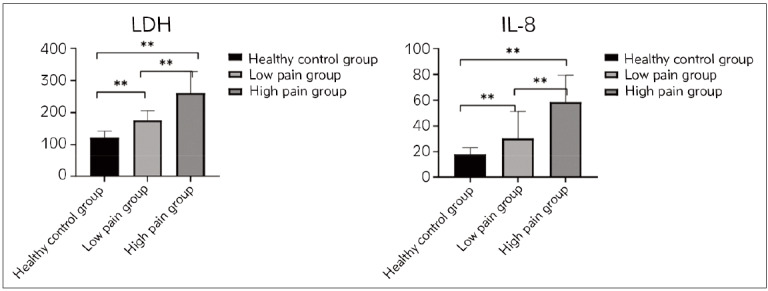
Serum LDH and IL-8 concentrations compared among three groups.

The serum LDH and IL-8 concentrations in HPG were (261.46±66.55) U/L and (58.53±20.91) pg/mL, respectively, which exceed LPG’s (P<0.05). Patients’ serum LDH in LPG was (175.84±29.88) U/L, and the concentration of IL-8 was (30.31± 20.91) pg/mL, which exceeded the healthy control group’s (*P*<0.05) in Figure 1.

### The relationship between serum LDH, IL-8, and patient pain grading

**Figure 2:**
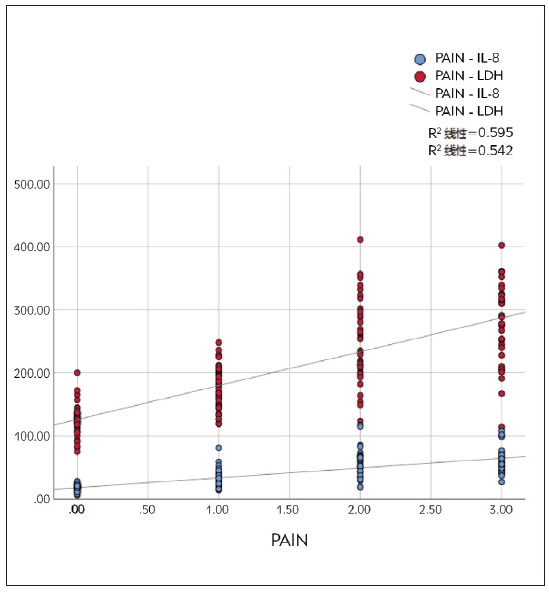
Trends in serum LDH and IL-8 levels in patients with different pain grades.

Pearson correlation analysis revealed a positive correlation between serum LDH concentration and pain grading (*r*=0.736, *P*=0.000). The serum IL-8 concentration and pain grading positively correlate (*r*=0.680, *P*=0.000). Serum LDH and IL-8 concentrations positively correlate (*r*=0.589, *P*=0.000). From Figure 2, serum LDH and IL-8 gradually increase as pain grade increases.

### Binary logistic regression results and predictive models for high pain

According to Table 2, LDH and IL-8 concentrations are independent risk factors for high pain levels (OR=1.033, 1.142, P<0.05). In the experiment, the logistic regression prediction model formula Y=constant+B1X1+B2X2+...+BnXn was used to set the joint diagnostic prediction model = -12.063+0.033×LDH+0.133×IL-8.

**Table 2:** Clinical data comparison.

Factor	B	SE	Wald	P	OR	95%CI
LDH	0.033	0.008	16.969	0.000	1.033	1.017~1.049
IL-8	0.133	0.008	21.948	0.000	1.142	1.080~1.207
Constant	-12.063	2.049	34.659	0.000	0.000	

### ROC analyzing

**Figure 3:**
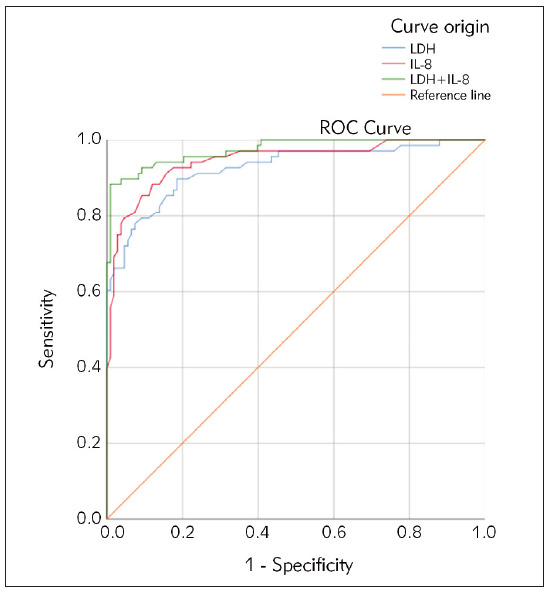
ROC curve with high pain.

The areas under ROC curves of LDH, IL-8, and predictive model (LDH+IL-8) in patients with high pain were 0.925, 0.945, and 0.974, respectively. The relevant standard for LDH is >190 U/L, the relevant standard for IL-8 is >36 pg/mL, and the relevant standard for the prediction model is >5.75. Please refer to Figure 3 for details.

## Discussion

The mechanism of PC pain is complex and is related to tumor infiltration, compression of peripheral nerves, inflammatory reactions, and bone metastasis [7] [8] [9] [10]. Cancer pain can affect patients’ quality of life, requiring accurate evaluation. Research has shown that elderly patients with digestive tract tumors still experience pain as the main symptom, accompanied by depression, anxiety, and even pain, which can lead to limited functional status [11]. At present, the diagnosis and treatment of PC patients mainly rely on palliative surgery, radiotherapy and chemotherapy, pain treatment, and mixed therapy. According to Aitken et al. [12], pain treatment accounts for 16.6% of PCs. Currently, visual simulation scoring methods [13] and digital scoring methods are mainly used to evaluate PC pain. However, these scoring methods are all subjective evaluations and impact feedback on the degree of pain. Pain, as the most common clinical symptom, is a complex physiological and psychological activity [14]. Ozcan et al. [15] showed that abdominal pain in PC patients is the most common symptom that affects quality of life. A good analgesic effect can improve the survival rate while worsening pain can reduce the patient’s survival rate. Effective pain treatment guided by serological indicators assists in prolonging the survival of metastatic PC patients. PC pain assessment should examine both the patient’s subjective score and the patient’s serological indicators.

The serological indicators related to pain are not yet clear. As an enzyme, LDH expression and activity significantly increase in PC cells due to excessive metabolism and increased energy demand. Lactic acid production in the intracellular matrix is related to cell proliferation and hypoxia. The increase in LDH activity in PC cells can promote metastasis and induce hypoxia, participating in pain exacerbation [16]. LDH can be regarded as a diagnostic and prognostic marker for primary pancreatic lymphoma [17]. Meanwhile, LDH has been found to be associated with multiple aspects, such as tumor growth and lymph node involvement [18]. High concentrations of LDH can stimulate angiogenesis, invasion, and metastasis of PC cells, expand tumor volume, compress or infiltrate surrounding tissues and nerves, and cause pain generation and aggravation. IL-8 is a cytokine that is highly expressed in the fibrotic environment of pancreatitis. It is an essential factor involved in the STAT 3/JAK2 pathway related to the inflammatory status of pancreatic ductal adenocarcinoma [19]. IL-8 can promote the recruitment and activation of tumor-related bodies, releasing inflammatory and pain mediators [20] [21]. In addition, IL-8 can directly act on surrounding neurons, causing the generation and transmission of pain signals [22]. LDH and IL-8 are routine PC patient screening indicators and can assist in assessing pain severity.

The results of this study show that the concentrations of LDH and IL-8 are higher in the HPG thanin LPG and higher in LPG than in the healthy control group. This indicates that LDH and IL-8 are closely related to the generation and exacerbation of PC pain, and there is a dose-effect relationship. These results accord with Noh et al. [23]. LDH concentration is positively correlated with pain VAS score grading, with higher concentrations in the HPG. IL-8 promotes tumor angiogenesis and cell movement, significantly increasing in PC tissue and blood, and is associated with invasion and metastasis. This suggests that LDH is involved in the exacerbation of PC pain and can be used as an evaluation indicator. IL-8 is involved in the generation and exacerbation of PC pain and can serve as a biological indicator of pain severity and changes. This study conducted logistic regression and predictive models and found that LDH and IL-8 concentrations were independent risk factors for high pain levels (OR=1.033, 1.142, *P*<0.05). Joint diagnostic prediction model =-12.063+ 0.033×LDH+0.133×IL-8. The areas under the ROC curves of LDH, IL-8, and predictive model (LDH+IL-8) in patients with high pain were 0.925, 0.945, and 0.974, respectively. It is suggested that combining the detecting methods of LDH and IL-8 can determine pain severity and trend better. Furthermore, relevant threshold values were proposed to determine the severity of pain and guide clinical treatment. LDH and IL-8 connect to tumor invasion closely and participate in tumor microenvironment and even pain generation, which can be used as assessment tools.

In summary, LDH and IL-8 are involved in the generation and development of PC pain, andchanges in concentration can reflect the severity of pain, which is expected to become indicators for pain assessment and prognosis. Combined applications can improve diagnostic accuracy and provide reference for pain treatment. The prediction model and related critical values constructed in this study can be used to assess the risk of pain exacerbation and the severity of pain and have guiding significance for the clinical diagnosis and treatment of PC pain. The widespread application of the model still requires expanding sample size validation. However, the research approach is innovative, and the results are relatively reliable, which has the hope of further transformation and application.

## Dodatak

### Conflict of interest statement

All the authors declare that they have no conflict of interest in this work.
